# A Case of Tuberculosis-Associated Acute Disseminated Encephalomyelitis in a Seven-Month-Old Infant

**DOI:** 10.7759/cureus.16299

**Published:** 2021-07-10

**Authors:** Jennifer H Yang, Kim T Vuong, Amaran Moodley, Nathaniel A Chuang, Dillon Y Chen

**Affiliations:** 1 Pediatric Neurology, Rady Children's Hospital, San Diego, USA; 2 Pediatric Neurology, University of California San Diego, San Diego, USA; 3 Pediatrics, University of California San Diego, San Diego, USA; 4 Pediatrics, Rady Children's Hospital, San Diego, USA; 5 Pediatric Infectious Diseases, University of California San Diego, San Diego, USA; 6 Infectious Diseases, Rady Children's Hospital, San Diego, USA; 7 Radiology, University of California San Diego, San Diego, USA

**Keywords:** tb – tuberculosis, demyelinating neurological disorder, white matter changes on mri, active pulmonary tuberculosis

## Abstract

A seven-month-old previously healthy female infant presented with acute onset encephalopathy and left focal weakness in the setting of three months of non-productive cough. She was diagnosed with pulmonary tuberculosis (TB), and neuroimaging showed multifocal non-enhancing T2 hyperintensities in the brain and longitudinal T2 hyperintensity in the spinal cord consistent with acute disseminated encephalomyelitis (ADEM). However, her cerebrospinal fluid (CSF) did not show evidence of TB infection. She was treated with high-dose steroids for five days with a steroid taper along with antitubercular medications with a remarkable recovery in gross motor function. This case suggests a previously unreported association between TB and an immune-mediated demyelinating syndrome in children that is clinically distinct from other more common forms of TB-associated central nervous system (CNS) complications.

## Introduction

Mycobacterium tuberculosis (TB) infection, both active and latent disease, affects about one-fourth of the world's population. In the United States, active TB disease has an incidence of 2.8 cases per 100,000 persons. In 2018, a total of 9025 cases of TB were reported, and an estimated 13 million people have latent TB [1]. About one percent of the active disease involves the central nervous system (CNS), typically manifesting as meningitis, and is associated with significant morbidity and mortality [2,3]. Acute disseminated encephalomyelitis (ADEM) is a demyelinating disorder of the CNS with an incidence between 0.3-0.6 per 100,000 per year [4]. The pathophysiology is an immune-mediated process often following an acute infection-causing perivenular demyelination and inflammation [4]. The median age of presentation for ADEM is five to eight years with a male predominance and is rarely reported in infancy [4]. ADEM is typically a monophasic disorder that causes multifocal neurological symptoms with distinct neuroimaging findings although recurrent disease can occur [4]. There is limited information regarding the relationship between TB and ADEM with only adult case reports published [5,6] and no cases reported in the pediatric population.

## Case presentation

A seven-month-old girl presented to an outside emergency room with four days of poor oral intake, fussiness, encephalopathy, and unusual movements in the setting of three months of intermittent non-productive cough. Her past medical history was significant for premature birth at 35 weeks gestation without any reported perinatal complications, developmental delay, or regression. She received the hepatitis B vaccine at birth, and she did not receive the bacille Calmette-Guerin (BCG) vaccine. She had bronchiolitis at two months of age and a non-productive cough since four months of age that was not previously investigated. There was no family history of seizures, developmental delay, autoimmunity, or immunodeficiency.

Vital signs on admission were significant for a temperature of 100.4 degrees Fahrenheit. Her physical exam demonstrated general irritability, neck rigidity with fixed left gaze preference, and left arm and leg hypertonicity. Given these acute neurological findings, the patient was transferred to the pediatric intensive care unit for higher-level management. Notably, her mother concurrently presented to an adult hospital with fever, chills, and cough and was diagnosed with acid-fast bacilli (AFB) smear-positive cavitary pulmonary tuberculosis soon after admission.

**Figure 1:**
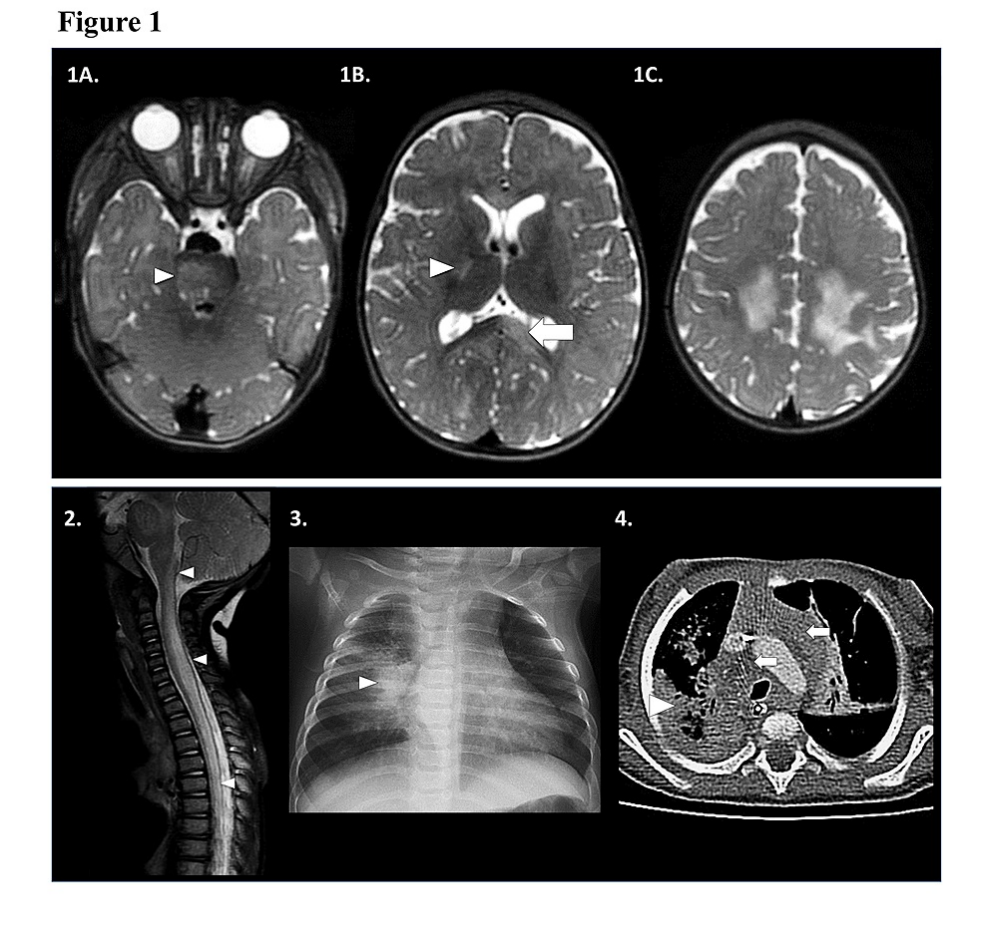
Neuroimaging and chest imaging 1) MRI brain, transaxial T2-weighted images showing multifocal T2-hyperintense foci in the: (A) right pons [white arrowhead]; (B) posterior limb of the right internal capsule [white arrowhead] and left splenium of the corpus callosum [white arrow]; (C) bilateral centrum semiovale and frontoparietal subcortical white matter. Other MRI sequences not shown revealed no associated reduced diffusivity, blood products, or contrast enhancement. 2) MRI spine, sagittal T2-weighted image showing extensive bilateral intramedullary T2-hyperintense edema and swelling along the cervicomedullary junction and throughout the cervical, upper and visualized mid thoracic spinal cord. No associated contrast enhancement was present on other MRI sequences. 3) Frontal chest radiograph: right perihilar opacity and hilar lymphadenopathy [white arrowhead]. Additional right upper, and left lower pneumonia and left hilar lymphadenopathy present. 4) Transaxial contrast-enhanced CT chest: dense necrotic right lower lobe [white arrowhead] with additional right middle and left upper lobe pneumonia, and bilateral bulky mediastinal lymphadenopathy [white arrows]. There was no enhancement or reduced diffusion.

Due to the patient’s respiratory symptoms, a nasopharyngeal swab was obtained which revealed rhino/enterovirus by polymerase chain reaction (PCR). Her initial blood tests were significant for a leukocytosis of 24 (10x3/uL) and an elevated C-reactive protein at 2.30 mg/dL suggesting active inflammation. An initial chest radiograph showed a right lung infiltrate that was concerning for pulmonary TB (Figure 1).

Combined with her encephalopathy and focal neurological symptoms, TB meningitis was highly suspected. A lumbar puncture at the outside facility revealed cerebrospinal fluid (CSF) pleocytosis (white blood cell count 101 cells/uL with 74% monocytes and 26% neutrophils), normal glucose (57 mg/dL), and elevated protein (120 mg/dL) with an unknown opening pressure. A repeat CSF analysis on admission confirmed an opening pressure of 32 cmH2O, pleocytosis with 17 white blood cells per uL, 0 erythrocytes, normal glucose (52 mg/dL), elevated protein (102 mg/dL), and normal lactate (1.7 mmol/L). The CSF AFB stain, culture, and mycobacterium tuberculosis PCR tests were all negative, but the serum interferon-gamma release assay (Quantiferon Gold) was positive. The remainder of her infectious workup is summarized in Table 1.

**Table 1:** Summary of diagnostic evaluations MRSA: Methicillin-Resistant Staphylococcus aureus; CSF: Cerebrospinal Fluid; PCR: Polymerase Chain Reaction; HSV: Herpes Simplex Virus; AFB: Acid-Fast Bacilli; MTB: Mycobacterium tuberculosis complex

Infectious	Bacterial MRSA nasal screen: negative CSF aerobic and anaerobic cultures: negative Blood aerobic and anaerobic cultures: negative Mediastinal tissue culture: negative Viral CSF meningoencephalitis PCR negative (includes Escherichia coli K1, Haemophilus influenza, Listeria monocytogenes, Neisseria meningitidis, Streptococcus agalactiae, Streptococcus pneumoniae, cytomegalovirus, cytomegalovirus, enterovirus, herpes simplex virus 1 and 2, human herpesvirus 6, human parachovirus, varicella zoster, Cryptococcus neoformans) CSF HSV 1 and 2 PCR: negative CSF enterovirus PCR: negative CSF West Nile Virus PCR: negative Serum West Nile IgG/IgM: negative HIV 4th generation antigen/antibody: negative Respiratory pathogen PCR panel: rhinovirus/enterovirus positive (includes adenovirus, coronavirus 229E, HKU1, NL63, OC43, human metapneumovirus, influenza A, A H1, A H1-2009, A H3, Influenza B, parainfluenza 1-4, respiratory syncytial virus A and B, Chlamydia pneumoniae, Mycoplasma pneumoniae) Fungal CSF Cryptococcus Ag: negative CSF Coccidioides Ab: negative Serum Coccidioides Ab: negative CSF fungal culture: no growth Mediastinal tissue culture: no growth Mycobacterial CSF AFB stain & culture: no growth, AFB smear-negative CSF MTB PCR (8/22/19): negative Quantiferon gold: positive Mediastinal tissue culture: positive for mycobacterium tuberculosis complex Next generation sequencing (NGS) plasma test: low positive for MTB complex PPD: positive at 10mm
Imaging	CT chest and abdomen w/ contrast: extensive pulmonary consolidation with necrotizing pneumonia and diffuse lymphadenopathy MRI brain with and without contrast MRI entire spine with and without contrast MR angiogram and MR venogram brain: normal
Pathology	CSF cytology: no malignant cells Mediastinal mass biopsy: necrotizing granulomas, AFB-positive and MTB complex PCR-positive
Genetic and metabolic	Whole genome sequencing (single): normal CSF amino acids: moderately elevated glycine
Electroencephalogram	Diffuse slowing; no epileptiform discharges
Audiology	Brain auditory evoked response: mild peripheral abnormality on the left consistent with middle ear function

To better characterize the extent of the TB infection, a computed tomography (CT) of the chest, abdomen, and pelvis with contrast was obtained, demonstrating extensive pulmonary consolidation with areas of non-enhancement suspicious for necrotizing pneumonia, and diffuse mediastinal, hilar, and supraclavicular lymphadenopathy (Figure 1). There were no additional findings in the abdomen or pelvis. A biopsy of the mediastinal mass revealed AFB-positive necrotizing granulomas, and cultures were positive for Mycobacterium tuberculosis complex (MTB), confirming the diagnosis of pulmonary tuberculosis with susceptibility to isoniazid, rifampin, pyrazinamide, and ethambutol. Her Mantoux skin test was also positive at 10 mm. Next-generation sequencing was performed in the serum to identify alternative pathogens, and only the MTB complex was identified.

Magnetic resonance imaging (MRI) of the brain and spine with contrast demonstrated multifocal non-enhancing T2 hyperintensities in the bilateral cerebral and cerebellar white matter, brainstem, and the spinal cord between the cervicomedullary junction and T10 spinal level, and no evidence of basilar meningeal involvement. It was noted that her MRI brain did not have basilar enhancement with contrast-enhancing exudates, which are often seen in TB meningitis. Given the extensive brain abnormality, a continuous eight-lead bedside electroencephalogram (EEG) was obtained, which did not show any epileptiform discharges or subclinical seizures. Rapid whole-genome sequencing was performed to look for possible other metabolic and genetic conditions given the atypical MRI brain findings. However, no pathogenic mutations or variants of unknown significance were identified.

Due the initial concern for CNS TB infection, she was started on dexamethasone 0.6mg/kg/day and four-drug TB therapy consisting of isoniazid (INH) 9 mg/kg/day, rifampin 18 mg/kg/day, pyrazinamide (PZA) 13 mg/kg/day and streptomycin 21 mg/kg/day. However, after one week of treatment, rifampin, INH, and PZA were held due to liver toxicity, and she was switched to ethambutol 18 mg/kg/day and levofloxacin 10 mg/kg/day. Rifampin was eventually restarted four days later, and INH was restarted two weeks later. One week into the hospitalization and treatment with antitubercular medications, her clinical condition, and neurological exam remained unchanged. A repeat MRI brain showed evolving and more conspicuous multifocal non-enhancing areas of T2 hyperintensity in the cerebral and cerebellar white matter, brainstem, and spinal cord (Figure 1) most compatible with an acute demyelinating process.

Her clinical course and MRI brain findings suggested a neuroinflammatory process rather than an active infectious process. The patient was then treated with high dose methylprednisolone 30 mg/kg daily for five days, which significantly improved her overall mobility and right-sided neglect [7]. In addition, she was treated with streptomycin, rifampin, INH, and ethambutol during the two-month intensive phase followed by rifampin and INH during the continuation phase to complete a total 12-month TB treatment course. Since she had a robust response to pulse steroids, she was given an eight-week prednisone taper to prevent further relapses. The patient was subsequently transferred to inpatient physiatry where she received two months of rehabilitation.

At her neurology follow-up at 18 months of age, she had improved tone in the left upper extremities with residual hypertonicity and hyperreflexia in both lower extremities. Developmentally, she was cruising to walk, transferring objects with good eye contact, and babbling with appropriate social interaction. She was eating well without any signs of dysphagia. An MRI brain and spine obtained four months after her initial presentation demonstrated resolved white matter lesions, consistent with the typical course for monophasic ADEM.

## Discussion

When the patient first presented, TB meningoencephalitis was at the top of the differential. She was empirically treated with dexamethasone 0.6 mg/kg/day along with antitubercular treatments for TB meningitis while CSF studies were still pending. The steroid dosing was based on previously published data on the treatment of CNS TB meningitis in children [8]. Identification of M. tuberculosis in CSF can be challenging. For example, Kennedy and Fallon [9] recorded a sensitivity of microscopy and culture to be 37% and 52% respectively, while nucleic acid amplification tests (NAATs) such as PCR were reported to have an average sensitivity of 56% [9]. A meta-analysis evaluating newer NAATs reported a sensitivity of 64% and specificity of 98% [10]. While serial testing by repeated lumbar punctures up to four times improves the sensitivity considerably [11], CSF analyses often can confirm TB meningoencephalitis but cannot rule it out. In cases where clinical suspicion for TB meningoencephalitis remains high without confirmatory testing, invasive brain biopsy can be considered, especially in the setting of suspected CNS tuberculoma. 

The failure to respond to initial four-drug antitubercular therapy with low-dose dexamethasone suggested an alternative etiology of her neurologic symptoms and prompted a reassessment of her symptoms. Indeed, the repeat MRI demonstrated more conspicuous multifocal T2 lesions which are typically seen in acute demyelinating syndromes. Coupled with the patient’s encephalopathy, the findings were most suggestive of ADEM [4]. Historically, TB encephalopathy is a separate entity reported in the literature as a rare consequence of TB infection [3]. This is based on a report on the pathology of TB encephalopathy describing findings of microvascular necrosis, perivascular macrophage reaction with demyelination, focal glial nodules, and occasional hemorrhagic lesions [12]. However, there is limited literature on further characterization of this entity, suggesting that TB encephalopathy may be a descriptive term for a demyelinating syndrome not recognized at the time.

Our patient met the clinical criteria for ADEM. The definition of ADEM has been revised over the years with certain core features. The International Pediatric Multiple Sclerosis Study Group defined ADEM as an acute or subacute attack of a CNS demyelinating disease with multifocal neurologic symptoms and encephalopathy seen more commonly in the pediatric population [13]. Pohl et al. [4] further characterized this as a multifocal syndrome of presumed demyelinating etiology with associated encephalopathy, MRI abnormalities consistent with a demyelinating process, and resolution of MRI findings after three months. These attacks can be monophasic or relapsing and are historically seen as a post-infectious phenomenon especially for the monophasic disease [4]. CSF studies typically show mild pleocytosis and elevated protein [4]. More recently, myelin oligodendrocyte (MOG) antibody-associated disease has been recognized to comprise a subset of patients with ADEM presenting with confluent white matter changes, seizures, and longitudinal spinal cord lesions [14]. In fact, anti-MOG antibodies have been found in up to 33-66% of ADEM cases, and anti-MOG disease is responsive to either high-dose corticosteroids or intravenous immunoglobulins, though the latter seems to be overall more effective [4,15]. Multiple sclerosis can be considered as it can present with neuroimaging findings similar to ADEM in the pediatric population. However, given the patient’s age and the resolution of imaging findings, ADEM was thought to be more likely. A metabolic white matter disease such as leukodystrophy was also considered, but this was unlikely given negative whole-genome sequencing, the dramatic response to steroids, and lesion improvement on subsequent MRI. 

Interestingly, our patient was rhino/enterovirus PCR positive in a nasopharyngeal sample, but enterovirus was not detected in the CSF. This is important as enteroviruses can also cause a range of infectious and postinfectious neurological complications. However, there is also limited literature on ADEM triggered by enteroviruses. Prior studies include a case series reporting four pediatric patients with enterovirus encephalitis who had MRI radiological findings consistent with ADEM [16]. Two of the four cases had a biphasic course in which the patients recovered from the initial enterovirus encephalitis but had a clinical relapse with radiological evidence of ADEM [16]. Unlike our patient, all four patients were tested positive for enterovirus PCR in the CSF at the time of ADEM diagnosis, including one patient whose PCR was initially negative. It is possible that our patient reflects a case of PCR-negative enterovirus-related ADEM. ADEM has also been associated with other CNS infections such as Haemophilus influenzae [17], Mycobacterium intracellulare [18], and varicella-zoster [19].

Herein, we present a pediatric case of TB infection with associated encephalitis and myelitis with neuroimaging most consistent with an acute demyelinating process. We determined that ADEM was more likely given: 1) repeated CSF testing with negative AFB stain, culture, and PCR testing, 2) MRI brain findings more consistent with ADEM and not that of TB meningoencephalitis or vasculitis, 3) no TB meningoencephalitis associated complications such as hydrocephalus or periventricular infarctions [20], 4) initial failure to respond to the antitubercular therapy, and 5) the evolution of her MRI brain following treatment. This case was clinically interesting since ADEM is rarely reported in infancy, and to our knowledge, there have been no reports of young infants presenting with ADEM in the setting of TB disease. While this is not the first report of TB-related ADEM [5,6], this is the first reported pediatric case. There are limitations to this case report. Unfortunately, anti-MOG antibody testing was not sent from our patient at the time of evaluation, and further tissue investigations such as a brain biopsy were not pursued. While brain biopsies should be considered in clinically difficult cases, it is an invasive procedure, and it was not pursued once the patient showed good clinical improvement on high-dose steroid treatment. 

## Conclusions

The diagnosis of ADEM should be considered in a child with multifocal neurological symptoms, pulmonary TB, and associated evidence of demyelination on MRI. Early recognition and treatment with high-dose steroids may greatly improve clinical outcomes. It is important to investigate evidence of TB meningoencephalitis as well as other etiologies for CNS infection as other viral infections can cause encephalitis with similar neuroimaging findings. Repeat imaging should be performed after three to four months to confirm that the lesions are resolved.
